# Cardiac commitment driven by MyoD expression in pericardial stem cells

**DOI:** 10.3389/fcell.2024.1369091

**Published:** 2024-03-27

**Authors:** Jianfeng Zhao, Limei Rui, Weili Ouyang, Yingcai Hao, Yusong Liu, Jianfeng Tang, Zheheng Ding, Zenghui Teng, Xueqing Liu, Hongtao Zhu, Zhaoping Ding

**Affiliations:** ^1^ Department of Cardiology, The People’s Hospital of Danyang Affiliated to Nantong University, Danyang, China; ^2^ Institute of Biochemistry and Molecular Biology II, Heinrich-Heine University of Düsseldorf, Düsseldorf, Germany; ^3^ Institute Neuro and Sensory Physiology, Heinrich-Heine University of Düsseldorf, Düsseldorf, Germany; ^4^ Institute of Molecular Cardiology, Heinrich-Heine University of Düsseldorf, Düsseldorf, Germany

**Keywords:** pericardium, myogenic commitment, MyoD, stem cell therapy, myocardial infarction, myogenic progenitors

## Abstract

Cellular therapy holds immense promise to remuscularize the damaged myocardium but is practically hindered by limited allogeneic sources of cardiac-committed cells that engraft stably in the recipient heart after transplantation. Here, we demonstrate that the pericardial tissue harbors myogenic stem cells (pSCs) that are activated in response to inflammatory signaling after myocardial infarction (MI). The pSCs derived from the MI rats (MI-pSCs) show *in vivo* and *in vitro* cardiac commitment characterized by cardiac-specific Tnnt2 expression and formation of rhythmic contraction in culture. Bulk RNA-seq analysis reveals significant upregulation of a panel of genes related to cardiac/myogenic differentiation, paracrine factors, and extracellular matrix in the activated pSCs compared to the control pSCs (Sham-pSCs). Notably, we define MyoD as a key factor that governs the process of cardiac commitment, as siRNA-mediated *MyoD* gene silencing results in a significant reduction of myogenic potential. Injection of the cardiac-committed cells into the infarcted rat heart leads to long-term survival and stable engraftment in the recipient myocardium. Therefore, these findings point to pericardial myogenic progenitors as an attractive candidate for cardiac cell-based therapy to remuscularize the damaged myocardium.

## Introduction

Adult cardiomyocytes (CMs) have little regenerative capacity to replenish the lost myocytes after ischemic injury, known as myocardial infarction (MI), and the damaged tissue is ultimately replaced by fibrotic noncontractile scar tissue (Laflamme and Murry, 2011). In the last 2 decades, cellular therapy has emerged as a promising approach to remuscularize the damaged heart by implanting a wide variety of autologous and allogeneic mesenchymal stem cells (MSCs) derived from multiple stromal sources (Sanganalmath and Bolli, 2013), including cardiac-specific stem cells derived from the heart itself (Vicinanza et al., 2017). This strategy, while promising, has been hindered by marginal improvement of cardiac function (Perin et al., 2023), in part due to low engraftment and persistence of the transplanted cells in the harsh environment and negligible direct differentiation into cardiomyocytes (Li et al., 2021).

Alternatively, differentiated CMs derived from allogeneic pluripotent stem cells, such as embryonic stem cells (ESCs) or induced pluripotent stem (iPS) cells, appear to be the most appropriate source strategy (Menasché, 2023). Cardiac-committed cells are found to stably engraft in the recipient heart together with significant improvement in contractile function (Chong et al., 2014; Shiba et al., 2016). The favorable engraftment of CMs implies that, in contrast to multipotent MSCs, cells that have been induced to cardiac commitment might be harnessed with a unique capacity to survive and persist in the recipient heart, likely through mechanisms related to a profound alteration of the molecular network after lineage specification (Menasché, 2023). The ability of robust engraftment, therefore, has sequentially advanced CM-based therapy into clinical trials (Trounson and McDonald, 2015), although some concerns must be overcome before their practical use in patients, including cardiac arrhythmia, immune rejection, and a potential risk of tumorigenesis (Anderson et al., 2014).

We have previously demonstrated that pericardial tissue harbors a stem cell pool, known as pSCs, that, trained by inflammatory signaling, evolved into a specialized population with boosted reparative activity and myogenic potential (Tang et al., 2018; Zhu et al., 2022; Zhu et al., 2023). However, we found that the cardiac benefits of the transplanted pSCs were solely mediated by paracrine factors released, whereas the pre-committed cells were poorly retained in the heart and negligibly converted into cardiomyocytes *in vivo* (Tang et al., 2018; Zhu et al., 2022). To this end, it is of particular interest to determine whether pSCs after cardiac commitment are able to survive and stably engraft in the recipient heart as well as the differentiated CMs derived from pluripotent stem cells.

In addition, it is equally important to define key hallmarks in the myogenic trajectory of the activated pSCs coaxed by inflammatory signaling (Tang et al., 2018). The inflammatory niche is a specialized microenvironment in which instructive cues are set up to trigger activation and proliferation of stem/progenitor cells in order to restore homeostasis, as seen in the bone, skin, and skeletal muscle, among others (Machado et al., 2021). In the activated pSCs, however, the early transcriptional response to inflammatory signaling and regulatory roadmap of myogenic commitment remains poorly defined.

Here, we employed bulk RNA-seq analysis to compare the transcriptional profile of the activated pSCs derived from the infarcted rats and defined myoblast determination protein 1 (MyoD) as a critical factor in regulating tissue-specific transcription during myogenic progression. Importantly, we revealed that transplantation of the cardiac-committed pSCs into the infarcted rat yields long-term engraftment in the recipient heart. Our results, therefore, point to allogeneic pSCs as an attractive candidate in cardiac cell-based therapy to overcome the aforementioned key limitations caused by iPS cells or ESC-derived CMs.

## Materials and methods

### Animal experiments

All the experiments were approved by the Institutional Animal Care and Use Committee at Nantong University (NTU, S20200410-003) and conducted in accordance with standard operating guidelines for animal care. Male Wistar rats (250–300 g of body weight) used in the present study were bred at the Animal Center of NTU, fed with a standard chow diet, and received tap water *ad libitum*.

Experimental MI was induced by transiently occluding the left anterior descending (LAD) coronary artery as previously described (Tang et al., 2018). In brief, animals were intubated and ventilated mechanically with a gas mixture (40% O_2_ + 60% N_2_) containing 1.5% v/v isoflurane (Shandong Keyuan, China). Surgical procedures were performed on a pre-warmed operating plate (37°C) with a pair of electrodes linked to an electrocardiograph (ECG, lead II, ADInstrument). Thoracotomy was carried out with a lateral cut along the left side of the sternum to identify the LAD via the visible landmarks on the heart surface. A polypropylene suture (6-0 Prolene^®^, Ethicon) with a tapered needle (P-3, 13 mm in length) was passed underneath the LAD marginally below the tip of the left auricle and firmly tied off to stop the blood flow downstream of the ligation site. Successful occlusion was confirmed by both visual inspection of myocardial blanching and immediate ST elevation in ECG registration. The knot was released 60 min after occlusion, and coronary perfusion resumed. The chest was then closed with one layer through the muscle and a second layer through the skin. Animals were weaned from ventilation and placed in a warm, oxygen-enriched environment until physical activity fully recovered.

Intramyocardial cell transplantation was performed in approximately 10 min after reperfusion was initiated. In each animal, a total number of 1 × 10^6^ cardiac-committed cells were suspended in 150 µl PBS and slowly injected into the three sites of the infarct border zone (3 × 50 µl) using a U-100 insulin syringe (29G, Micro-Fine, BD). No animals received immune suppressive agents in the entire follow-up period.

### Isolation of pericardial stromal cells

Pericardial stem/stromal cells (pSCs) were isolated from either the sham-operated (Sham) or the infarcted rats 5 days post-MI as previously reported (Tang et al., 2018). In brief, rats were sacrificed by overdosing isoflurane and then transiently immersed in a 75% ethanol solution for whole-body disinfection. Thereafter, the chest was opened under sterile conditions, and the heart, including the surrounding pericardial tissue, was taken out of the thoracic cavity and transferred to a sterile beaker containing ice-cold PBS. The pericardial tissue was carefully separated from the heart surface and minced into small fragments (approximately 1 mm^3^). The tissue pieces were digested in a 15-ml Flacon tube containing 5 ml of digestion solution (84 U/mL of collagenase II, Biochrom, Beijing, China) at 37°C with gentle rotation at a rate of 30 spm. Digestion was stopped after 25 min by adding 2 ml fetal calf serum (FCS, Sigma), and the resultant cell suspension was spun down to remove cell debris and fat droplets. The cell pellet was then re-suspended in a basic medium containing low-sugar Dulbecco’s modified Eagle’s medium (DMEM, sugar = 1,000 mg/ml, Sigma) supplemented with 30% FCS, penicillin (100 U/mL), streptomycin (0.1 mg/ml), and glutamine (1 mM). Cells were then seeded in a density of 2 × 10^3^/cm^2^ in a T75 culture flask, and non-adherent cells were removed 2 h after initial plating. The adherent stromal fraction was then termed pericardial stromal/stem cells (pSCs). The primarily isolated pSCs were cultivated at 37°C with 5% CO_2_ for 3–5 days until sub-confluence and then passaged.

### Myogenic differentiation

Given the pre-emptive myogenic potential of pSCs, particularly in the MI animal (Tang et al., 2018), cardiac commitment was induced solely by lowering the FCS concentration in the culture medium. In brief, when the cultivated pSCs reached 90% sub-confluency, the growth concentration of FCS in the medium (30%) was changed into a mixture containing only 3% FCS +5% horse serum, while other compounds remained unaltered, as reported in a serum-free protocol (Chal et al., 2016). The differentiating medium was refreshed every 3 days up to 2 weeks while spontaneous rhythmic contraction was inspected. After myogenic induction, the electroactivity of the cardiac-committed pSCs was evaluated by a multiple electrode assay, and their myogenic fate was examined by a mean of immunostaining (cardiac-specific Tnnt2, see below).

### Electrophysiology

A multiple electrode assay (MEA, Multi Channel Systems, Reutlingen, Germany) was employed to assess the electrophysiological activity. In brief, MEA chips were plasma-cleaned and coated with fibronectin (50 μg/ml) for 1 h at 37°C. Cardiac-committed pSCs after myogenic induction were replated on a standard 60-electrode MEA chip equipped with titanium nitrate electrodes of 30 μm in size with a spacing of 200 μm. Field electroactivity was obtained using a MEA1060INV MEA amplifier (Multi Channel Systems), and data were recorded using a QT screen (Multi Channel Systems) and analyzed offline with a QT analyzer (Multi Channel Systems). A firing frequency (FF) baseline was performed in DMEM supplemented with 8% FCS in a thermostatic chamber at a temperature of 37°C.

Pharmacological responses were tested by adding isoproterenol (ISO, 1μM, Sigma), acetylcholine (ACh, 1μM, Sigma), or HCN-channel blocker (ZD7288, 0.1μM, MCE) into the culture medium and washing it out after 2 min. FF was continuously monitored at the basal condition, intervention, and washout phases.

### Histology and immunostaining

Cardiac samples, including the heart and pericardial layer, from the Sham and MI animals were prepared as tissue blocks and sliced at a thickness of 7 μm using a cryostat. The cryosections were dried and fixed with 1% paraformaldehyde for 10 min at room temperature. Thereafter, tissue slides were either subject to hematoxylin and eosin (H&E) staining as standard protocol or immunochemistry. For immunostaining, the slides were first blocked with 5% normal goat serum (NGS) for 1 h at room temperature and then incubated with monoclonal anti-MyoD (1:400, rabbit monoclonal, ZRB1452, Sigma-Aldrich) and anti-Tnnt2 (1:200, mouse monoclonal, 13-11, Thermo Fisher) overnight at 4°C. After washing three times with PBS, the slides were further incubated with fluorochrome-conjugated secondary antibodies (TRITC, 1:400, or FITC, 1:200, goat IgG, Jackson Lab) at room temperature for 2 h, and the nuclei were counterstained with 4′,6-diamidin-2-phenylindol (DAPI, Sigma). The slides were finally mounted with Prolong™ Gold (Invitrogen). Fluorescence images were acquired using fluorescence microscopy (Olympus MX61) operated with CellSence^®^ software. The cross-sectional area of the implanted pSCs and mature cardiomyocytes was computed by manually contouring the boundary of the selected cells in the H&E-stained slices by computer-aided planimetry using ImageJ (NIH, USA). The value was derived as pixel counts and converted to absolute area (µm^2^) after referring to the known length of the scale bar. The relative intensity of Tnnt2 expression in the transplants was calculated in the immunostained slices and presented as a percent intensity related to the host myocardium in the same section.

Immunostaining on cultured cells was carried out as previously described (Tang et al., 2018). In brief, freshly isolated pSCs or pSCs after myogenic induction were seeded on sterile coverslips (24 × 24 mm), placed in a six-well plate, and cultured for 3 days until they reached 80% sub-confluence. The coverslips were then fixed with 1% paraformaldehyde and permeabilized with 0.01% Triton for 10 min at room temperature. Primary antibodies, including monoclonal anti-MyoD (1:200, rabbit monoclonal, ZRB1452, Sigma-Aldrich) and anti-Tnnt2 (1:200, mouse monoclonal, 13-11, Thermo Fisher), were added to the slips and incubated at 4°C overnight in a moist chamber. After washing three times with 1% NGS-PBS buffer, the slips were incubated with a secondary antibody (TRITC- or FITC-conjugated goat IgG, 1:100) for an additional 60 min at room temperature. The slides were counterstained with DAPI, sealed with mounting medium, and processed similarly to the tissue slides described above.

### RNA-seq and bioinformatic analyses

Bulk RNA sequencing (RNA-seq) analysis was commercially commissioned to Beyotime Biotech (Shanghai, China) and performed as previously described (Zhu et al., 2023). In brief, total RNA was isolated from the cultured Sham-pSCs and MI-pSCs after myogenic induction using an RNeasy mini kit (QIAGEN, Germany) according to the manufacturer’s instructions. Purified RNA samples (500 ng) were used for reverse transcription and library construction. The cDNA samples were sequenced on the Illumina HiSeq PE150 to acquire a paired-end read (150 bp). The total number of reads sequenced, GC content, and overall base quality score were computed by using FastQC for raw data quality control. The differentially expressed transcripts (DETs) between the two populations (Sham-pSC vs. MI-pSC) were computed with the *DESeq2* package (version 1.20.0) supplied by R software (version 3.4.4) via a likelihood ratio test implemented in the *DESeq* package. Data were then subjected to functional enrichment analysis by STRING and KEGG software (version 10.5).

In order to visualize the DET genes between the Sham-pSC and MI-pSC populations, the upregulated genes in individual animals were presented as logarithmic fold changes in relation to the mean levels in the animals in the Sham-pSC group, which were set as a factor of 1.

### RNA interference of *MyoD* expression

We used mRNA interference to reduce the mRNA level of the *MyoD* gene and examine MyoD-mediated myogenic potential in the MI-pSCs. The *MyoD* siRNA was synthetic and target-specific 19-23 nt siRNA oligo duplexes designed to knock down *MyoD* expression (GeneID: 4654). Transfection of siRNA into cells was performed according to the manufacturer’s instructions. In brief, MI-pSCs were isolated and cultivated until cell density reached 60%–70% (3–4 days on cover slips). *MyoD* siRNA (0.1 μM, GenePharma, China) or scramble siRNA (negative control) and transfection reagent (Lipofectamine 2000; Invitrogen, USA) were separately diluted in serum/antibiotic-free DMEM. The transfection reagent/siRNA mixture was then added to the culture medium and incubated in a serum-free medium for 6 h. Thereafter, the transfection medium was replaced with standard DMEM, and myogenic induction was performed as mentioned above.

We used quantitative real-time PCR (RT-qPCR) to examine the mRNA level of the *MyoD* in the MI-pSCs after siRNA-mediated gene silencing. In brief, total RNA was isolated from the cells 48 h after siRNA transfection and converted to cDNA with a first-strand cDNA synthesis kit (Invitrogen, USA) according to the manufacturer’s instructions. RT-qPCR was performed in a StepOne PCR apparatus (Applied Biosystems) using *MyoD* (Rn00580555_m1, Thermo Fisher) primers and *GAPDH* (Rn01775763_g1, Thermo Fisher) as a housekeeping gene. The relative expression of *MyoD* was normalized to *GAPDH* and calculated using the 2^delta^ Ct value methodology. All RT-qPCR assays were performed in duplicate.

### Statistical analysis

Data were presented as mean ± standard deviation (SD). All data were checked using normal (Gaussian) distribution and the Shapiro–Wilk normality test. A Student’s t-test with Welch’s correction was used to compare the percentage of positive cells stained with MyoD and Tnnt2 between the Sham-pSC and MI-pSC populations. The pharmacological tests on firing frequency and expression level and the percentage of positive cells of MyoD and Tnnt2 after *MyoD* silencing were analyzed with one-way analysis of variance (ANOVA). Differences were considered significant at *p* < 0.05. The Prism software package (version 9.0) was used for statistical analysis.

## Results

### Formation of myogenic progenitors in the pericardial stromal cells

**FIGURE 1:**
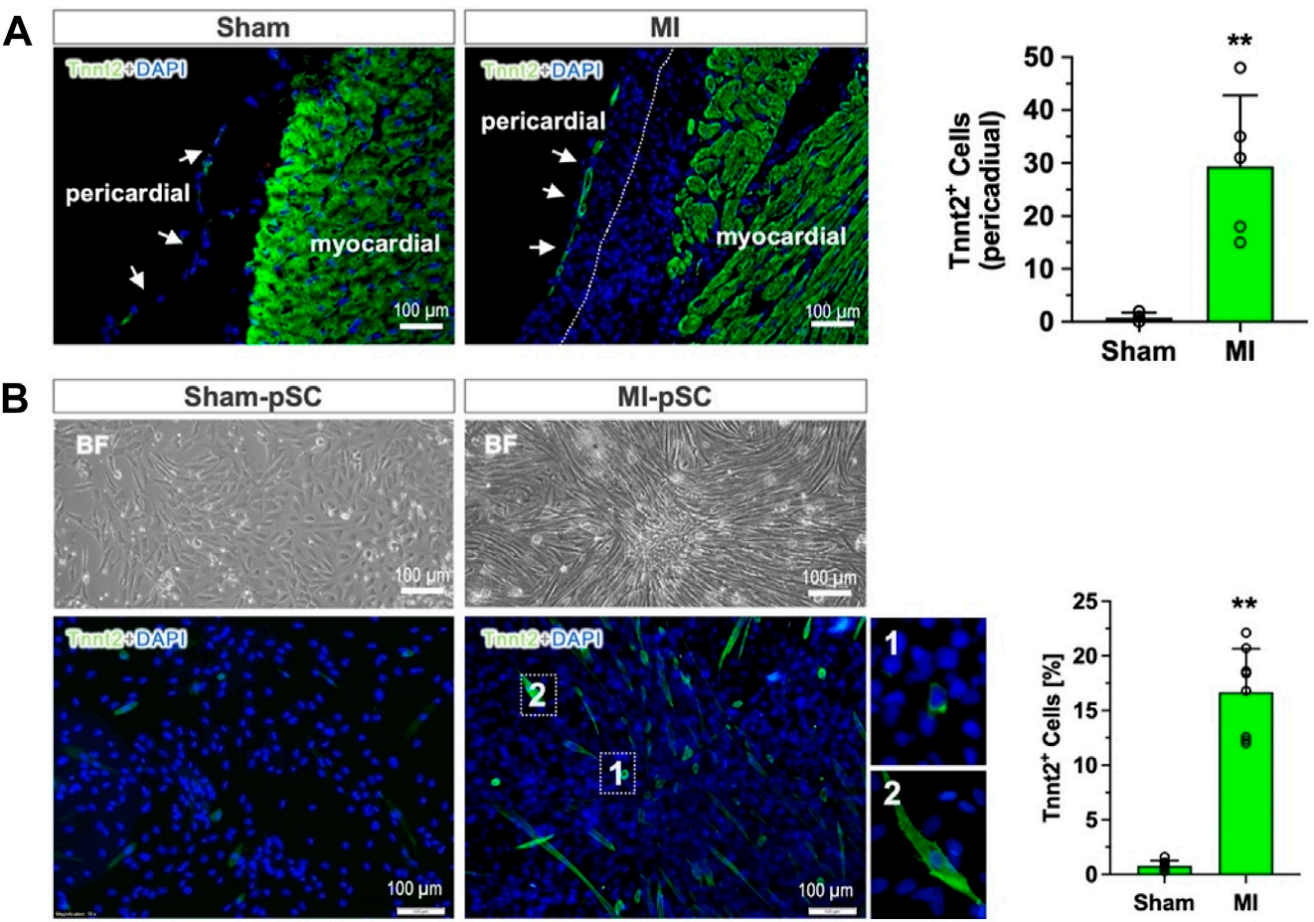
Myogenic differentiation of pericardial stem cells in *in vivo* and *in vitro* conditions. **(A)** The pericardial tissue was a thin monolayer covering the heart surface in the control heart (Sham). After MI, the number of cells increased, and epicardial cells grew together (dotted line). In the outermost layer of the pericardial tissue, a measurable number of Tnnt2-expressing cells were detected (approximately 30 cells per section, n = 6), while only a few were found in the Sham hearts (right panel, n = 4). **(B)** The isolated and cultured pSCs after myogenic induction (low-serum) showed morphological alteration in bright field (BF): maintaining a cobblestone shape in Sham-pSCs (n = 6) but becoming elongated and enlarged cell bodies in MI-pSCs (upper panel, n = 8), in which a significant number of Tnnt2-positive cells formed (15%, right panel). They showed two major subsets according to cell size: myoblast-like cells with small, round shapes (insert one in the lower panel) and enlarged cells with the striated morphology typically seen in cardiomyocytes (insert two in the lower panel). ** indicates *p* < 0.01.

Immunostaining of the heart section revealed that the pericardial layer was only a thin monolayer surrounding the heart surface in the Sham animals (Sham), but it expanded in cell numbers after MI, leading to a remarkable increase of the layer thickness to more than 100 µm. On some occasions, the pericardial tissue adhered onto the heart surface and grew together with epicardial cells (dot line in Figure 1A). Most interestingly, we found a measurable amount of Tnnt2 (cardiac-specific)-expressing cells formed in the outermost layer of the pericardial tissue, in contrast to the pericardial tissue in the Sham animals in which no Tnnt2-positive cells were detected (right panel in Figure 1A, *p* < 0.01).

To analyze the myogenic potential of pSCs, we isolated the stromal fraction from pericardial tissue and induced myogenic differentiation by a low-serum protocol. While the Sham-pSCs showed typical cobblestone morphology in culture after induction (left panel in Figure 1B), the MI-pSCs formed cell clusters with elongated cell bodies, and some of them developed spontaneous rhythmic contraction (Supplementary Video S1). Immunostaining revealed the existence of plentiful Tnnt2-expressing cells in MI-pSCs after myogenic induction, which, according to cell size, were categorized into subsets: myoblast-like cells with a small, round shape (insert one in the lower panel of Figure 1B) and enlarged cells with the striated morphology typically seem in cardiomyocytes (insert two in the lower panel of Figure 1B). As myogenic commitment was observed preferentially in the MI heart *in vivo* and *in vitro* conditions, our results suggest that inflammatory signaling after MI is a likely critical step for pSCs to gain myogenic potential.

**FIGURE 2:**
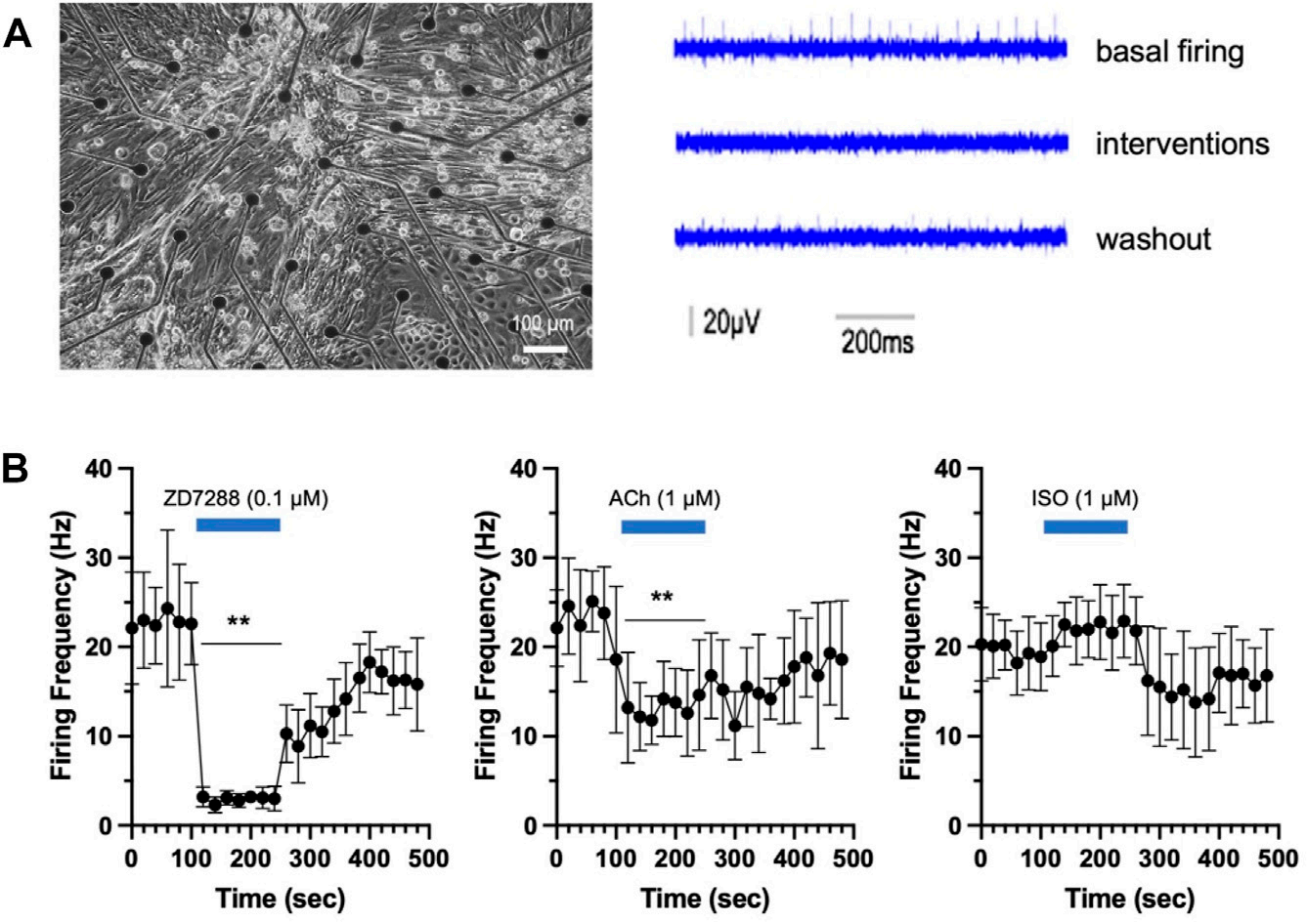
Electrical property of the cardiac-committed pSCs. **(A)** Field electroactivity of the cardiac-committed cells was recorded in the multichannel MEA system, and the firing frequency (FF) was analyzed in the basal, intervention, and washout phases. **(B)** The spontaneous FF was 20 Hz at basal conditions and was significantly reduced by the addition of either ACh (n = 4) or HCN blocker (ZD7288, n = 4), while treatment with ISO (n = 4) had minor changes. The negative chronotropic effects were partially reversible. ** indicates *p* < 0.01.

We then examined the electroactivity of the cardiac-committed cells by using the MEA system, which detects the extracellular electroactivity of cells on a pair of electrodes (left in Figure 2A). The spontaneous FF in the culture cells at basal condition was at a rate of 20 Hz (right in Figure 2A). Only a minor change in FF was found when cells were treated with ISO. Notably, the addition of either ACh or HCN blocker (ZD7288) yielded a significant FF reduction (*p* < 0.01, Figure 2B). The negative chronotropic effect was partially reversible as FF failed to fully restore in the washout phase (Figure 2B). The data suggest that cardiac-committed pSCs developed pacemaker-like features and were partially equipped with catecholamine/acetylcholine responses, similar to adult cardiomyocytes.

### Induction of myogenic genes in MI-pSCs

**FIGURE 3:**
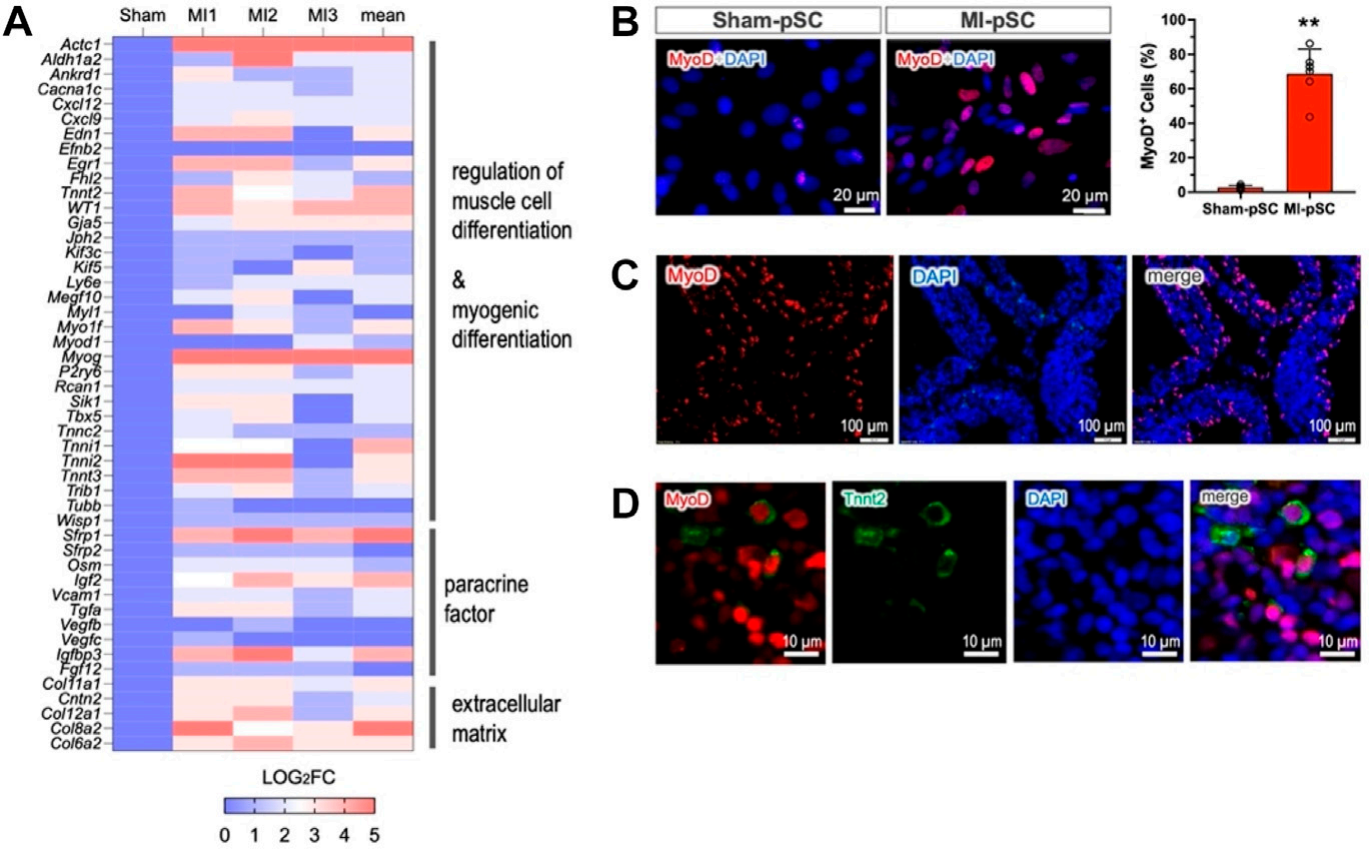
MyoD as a master regulator of myogenic commitment. **(A)** The selected genes obtained by bulk RNA-seq were visualized as the logarithmic fold change of individual animals (MI1-3, n = 3) in relation to the control value (referred to as factor 1, n = 3) and presented as a heatmap plot. The upregulated genes were annotated to muscle cell differentiation, paracrine factors, and formation of extracellular matrix. **(B)** Upregulation of *MyoD* (*Myod1*) was confirmed by immunostaining in the isolated pSCs. *MyoD* expression in MI-pSCs was found to be more intense and significantly increased the percentage of positive cells (n = 6) compared to the control pSCs, which showed only a few cells and weak expression (n = 5). **(C)** Abundant MyoD-positive cells were also readily detected in the tissue section of the MI heart (5 days). **(D)** A small fraction of MyoD/Tnnt2 double-positive cells (5.4%) in the pericardial layer suggested early cardiac commitment in *in vivo* conditions. ** indicates *p* < 0.01.

In order to explore potential factors that govern myogenic progression in MI-pSCs, we performed bulk RNA-seq analysis and compared DET genes between Sham-pSC and MI-pSC populations after myogenic induction. A total of 32,544 transcripts were identified; a total of 277 transcripts were found to be significantly upregulated and 209 downregulated in MI-pSCs. By performing gene annotation and functional enrichment, we found that the upregulated genes were mostly related to cardiac muscle tissue development (GO:0048738), regulation of muscle cell development (GO: 0054024), and cardiac cell differentiation (GO:0035051), suggesting a robust induction of myogenic potential in MI-pSCs. To visualize the DET particularly related to myogenic differentiation, the logarithmic fold change of individual animals (MI1-3, n = 3) in relation to the control value (referred to as factor 1, n = 3) is presented in a heatmap (Figure 3A). Notably, we found that in MI-pSCs, both cardiac (*Tbx5, Actc1, and Tnnt2*) and skeletal muscle (*Myog, Myf5, Tnni2, and Tnnt3*)-specific genes were broadly induced, indicating the formation of bipotent myogenic progenitors as to embryonic counterpart (Chan et al., 2016). In addition, the activated pSCs also showed the enhanced paracrine effects of a panel of trophic factors and increased formation of extracellular matrix, reflecting a general tissue response to inflammatory signaling with boosted reparative activity (Figure 3A).

As *MyoD* and its family members are critically involved in the process of myogenic commitment (Olson, 1993), we further verified *MyoD* expression in the cultured pSCs before myogenic induction. *MyoD* expression was found to be more intense, and the percentage of positive cells was significantly increased in the MI-pSC population compared to the control pSCs, which showed only a few positive cells and weak expression (Figure 3B, *p* < 0.01). Remarkably, we detected abundant MyoD-positive cells in the tissue sample of the pericardial layer of the rat 5 days after MI (42.3% ± 8.2%, n = 5, Figure 3C), suggesting early induction of *MyoD* expression. Interestingly, at this stage, a small fraction of MyoD-positive cells readily showed parallel Tnnt2 expression (5.4 % ± 3.4%, n = 4, Figure 3D).

**FIGURE 4:**
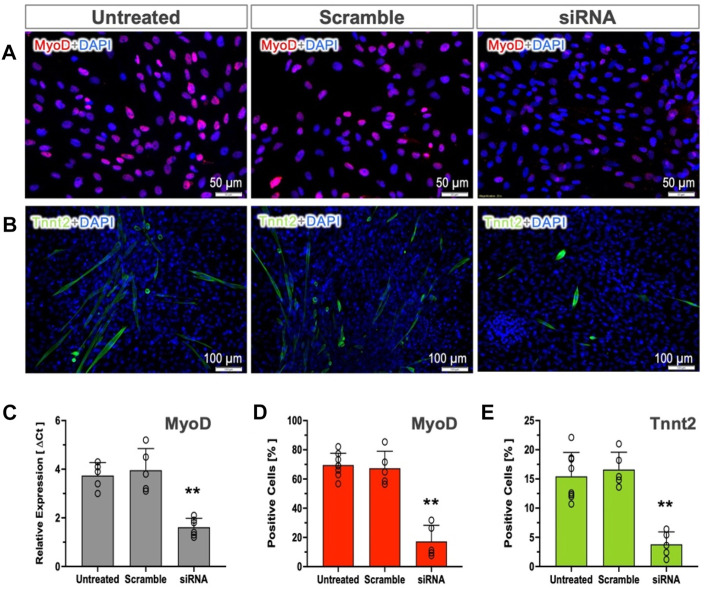
Role of MyoD in cardiac commitment. **(A, B)** MyoD and Tnnt2 expression was detected by immunostaining in the untreated or treated with Scramble control and targeting siRNA. **(C)** RT-qPCR analysis revealed that the mRNA level of the *MyoD* was not altered by Scramble siRNA treatment (n = 5) but significantly reduced by more than 50% in the targeting siRNA-treated pSCs (n = 6). **(D, E)** Quantitative analysis revealed that the cultured MI-pSCs in the untreated controls (n = 8) constituted approximately 60%–80% MyoD-positive and 10%–20% Tnnt2-positive cells, and the percentages were not altered in the Scramble siRNA-treated pSCs (n = 5). Silencing of *MyoD* expression (n = 5) led to a significant reduction in the percentage of positive cells after MyoD and Tnnt2 staining. ** indicates *p* < 0.01.

To test whether *MyoD* expression in pSCs is indispensable for myogenic commitment, we used siRNA-mediated silencing to knock down *MyoD* expression. The percentages of positive cells (MyoD and Tnnt2) and *MyoD* expression were not altered by transfection of scramble controls (Figure 4). The siRNA targeting *MyoD* in MI-pSCs led to a more than 50% reduction in *MyoD* mRNA level as compared to the untreated or scramble controls (Figure 4C), which corresponded to a significant reduction of MyoD- and Tnnt2-positive cells in the siRNA treated cells (Figures 4D,E, *p* < 0.01). These results indicate that MyoD plays an obligatory role in inducing pSCs towards myogenic commitment.

### Cardiac retention of cardiac-committed cells after transplantation

**FIGURE 5:**
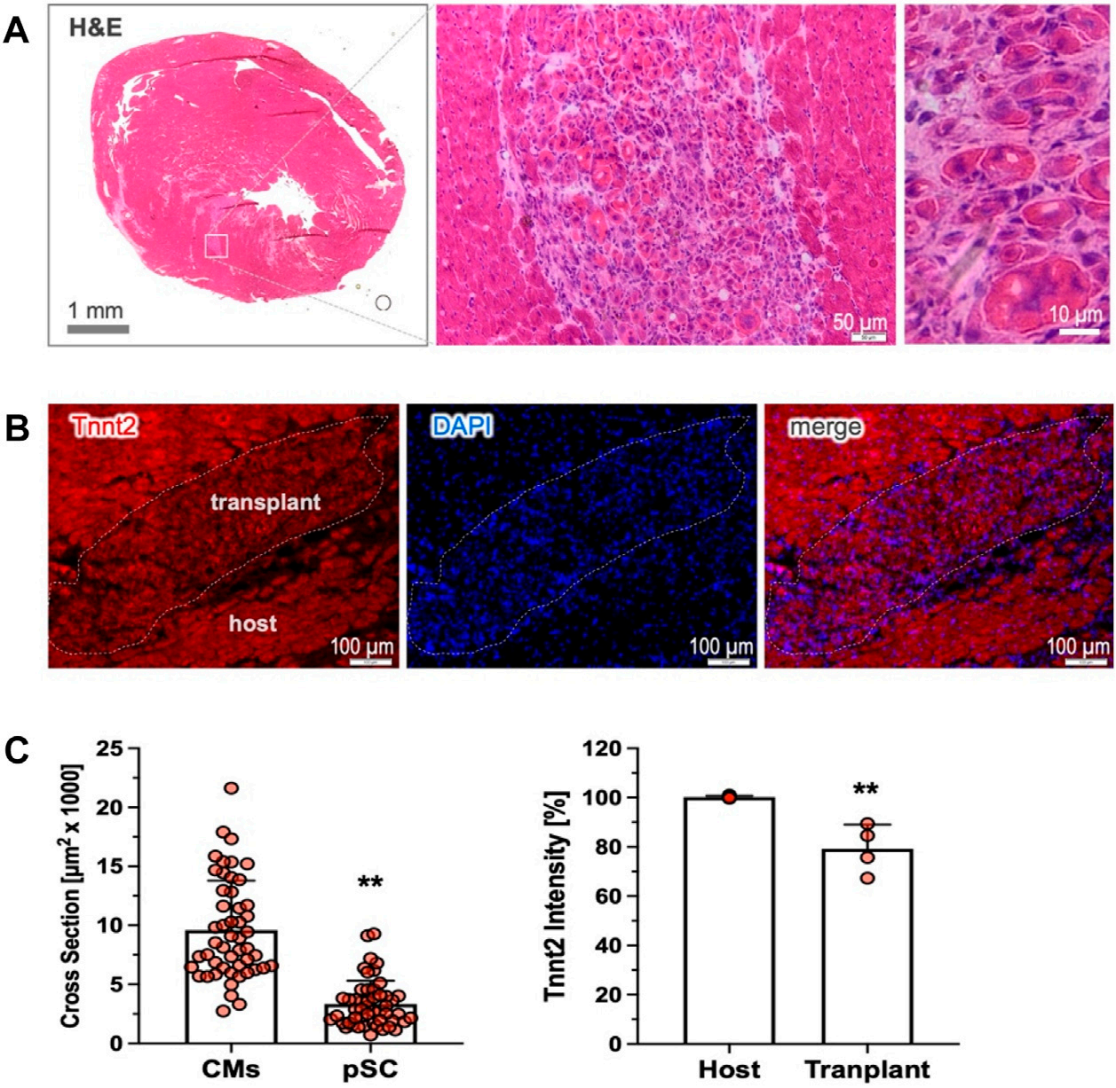
Cardiac retention of the cardiac-committed pSCs. **(A)** Cardiac transplants were detected by H&E staining in the recipient heart, shown as subtle clusters nestling the host tissue 4 weeks after transplantation (n = 4). The implanted cells were round, sharp, and relativity small in size compared to the host cardiomyocytes, and they were intermingled with multiple non-myocytes. **(B)** The engrafted cells exhibited intensively positive staining for Tnnt2, indicating the persistence of durable cardiac fate after transplantation. **(C)** Quantitative analysis revealed that the implanted cells were modest in the cross-sectional area (n = 50, left) and relatively weak in Tnnt2 expression (n = 4) compared to the mature cardiomyocytes (n = 50, and n = 4). ** indicates *p* < 0.01.

We have previously demonstrated that the pre-committed pSCs disappeared shortly after cardiac injection, leading to poor cardiac retention (Wang et al., 2016). We next explored whether the cardiac-committed pSCs could survive and stably engraft in the recipient heart. In the rat model of MI without immunosuppressive therapy, we found that intramyocardial injection of the induced cells led to the durable formation of subtle clusters nestling within the host tissue, as shown by the representative H&E staining (4 weeks, n = 4). The transplanted cells were round, sharp, and relativity smaller than the host cardiomyocytes (Figure 5C), and they were intermingled with multiple non-myocytes within the transplant (Figure 5A). Most importantly, the injected cells showed Tnnt2 expression as a marker of persistent cardiac lineage specification, although the intensity was relatively weak compared to the mature cardiomyocytes (Figure 5C). Therefore, this experiment provides “proof-of-concept” evidence showing that cardiac-committed cells have the ability to survive and persist in the host tissue after transplantation, although the issues of electrical integration and cardiac benefits need to be further explored.

## Discussion

In the present experiments, we demonstrate that the pericardial stem cells, in response to cardiac injury (MI), become progressively more dedicated in a trajectory towards cardiac lineage commitment in *in vivo* and *in vitro* conditions. In this process, upregulation of the *MyoD* gene is likely a key step to direct myogenic commitment. Injection of the cardiac-committed cells into the infarcted heart yields long-term survival and stable engraftment in the host myocardium. Therefore, these results propose that the myogenic progenitors from a pericardial source are an attractive candidate for cardiac cellular therapy to remuscularize the damaged myocardium.

### Myogenic progenitors in the epicardial tissue

The pericardium is a continuous layer of the parietal epicardium and consists of heterogeneous populations, including nerves, adipocytes, and vessel cells. Recent studies have disclosed that the pericardial layer harbors a stem cell pool that, in response to injury-associated signals, gains myogenic potential and reparative activity (Bollini et al., 2014; Tang et al., 2018). In the present experiments, we showed that cardiac commitment occurred in an *in vivo* scenario in which the pericardial layer expanded in cell numbers and adhered to the heart surface after MI (Figure 1A). After isolation, pSCs were demonstrated to be phenotypically identical to mesenchymal stem cells with minimal contamination of hematopoietic and endothelial lineages (Wang et al., 2014; Tang et al., 2018; Zhu et al., 2022). After myogenic induction *in vitro*, pSCs formed myoblast-like clusters and gave rise to cardiac lineage under low-serum cultivation. It is interesting to note that significant myogenic commitment was found in the MI-pSC, and much less extensively in the Sham-pSC, suggesting the inflammatory niche is an important activating signal to initiate myogenic progression (Tang et al., 2018).

We found that inflammatory signaling upregulated a panel of genes related to myogenic induction for both skeletal and cardiac specification (Figure 3A). In parallel, upregulation of both skeletal/cardiac genes largely mimicked the existence of bipotent anlage that leads to the formation of both the head muscles and the second heart field (SHF) during organogenesis (Chan et al., 2016). The pericardial stromal cells developmentally originated from the Mesp1^+^ derivatives in the cardio-pharyngeal mesoderm (CPM), also known as the lateral splanchnic mesoderm, that gave rise to bipotent progenitors to form the SHF and distal facial skeletal muscles (Lescroart et al., 2010). Thus, pSCs are developmentally the SHF progeny (Chong et al., 2011) and, in adulthood, probably still bear the genetic signatures of common progenitors to their embryonic counterparts.

### Myogenic induction by inflammatory cues

A transient increase in local proinflammatory cytokine expression, such as IL-1, IL-6, and TNF-α at the site of injury, is indispensable for activating tissue stem cells (Karin and Clevers, 2016; Godwin et al., 2017). Cytokines are found to bind the upstream enhancer of Pax7 that initiates the sequential expression of specific transcription factors, including Myf5, MyoD, myogenin, and MRF4, transcription factors collectively known as myogenic regulatory factors (MRFs) for myogenic progression (Buckingham and Rigby, 2014). This process is crucial in skeletal muscle to generate adequate myoblasts through satellite cells reentering the cell cycle and proliferating, and ultimately to repair the damaged myofibers through myogenesis (Fu et al., 2015).

In the damaged heart, acute proinflammatory signaling and immune cell recruitment to the site of infarction constitute the initial phase of the cardiac inflammatory response (Toldo and Abbate, 2017). The inflammatory signaling induces EPDC into an active state with myogenic potential to foster myogenesis in salamanders (Eroglu et al., 2022) or cardiac repair in a paracrine manner in mammals (Zhou et al., 2011). Notably, we have previously found cardiac inflammation in the MI heart was widespread beyond the injured site to the pericardial tissue surrounding the heart, leading to proliferation (Zhu et al., 2023) and, most importantly, in the present study, myogenic activation in pSCs (Figures 3D,E). This response is likely mediated by cardiac transudate, or pericardial fluid, in which abundant proinflammatory cytokines were previously detected (Tang et al., 2018).

### MyoD-mediated myogenic potential

MyoD is a family of members sharing a basic helix-loop-helix (HLH) motif (Olson, 1993) that essentially acts as a myogenic determination gene in the process of satellite cell activation (Wood et al., 2013). Genetic experiments revealed that ectopic expression of MyoD in dermal fibroblasts (Murry et al., 1996) or cardiac fibroblasts (Etzion et al., 2002) led to the conversion of fibroblasts into myogenic commitment, suggesting MyoD as a key molecule that induces myogenic progression (Hirai et al., 2013). In the present experiments, we found that *MyoD* expression in the quiescent pericardial cells was at a relatively low level but increased dramatically after myocardial infarction (Figure 3B). *MyoD* induction was associated with a global upregulation of multiple myogenic genes, including other members of the MyoD family (*Myog and Myf5*, Figure 3A). Notably, siRNA-mediated suppression of the *MyoD* gene significantly reduced the formation of Tnnt2-expression cells in MI-pSCs (Figure 4), suggesting that, in line with previous observations (Lee and June 2019; Mannino et al., 2022), MyoD may act as a master regulator of myogenic commitment in response to inflammatory cues.

*MyoD* expression is mainly upregulated by Hedgebog (Voronova et al., 2013) but also largely depends on the multiple levels of regulations. However, in many instances, the natural transcription factors do not induce a sufficiently robust response to completely reprogram the cell phenotype (Buckingham and Rigby, 2014). In our experimental MI setting, an intact pericardial sac seems to be necessary to converge the cardiac transudate with abundant inflammatory factors (Tang et al., 2018). In this context, MyoD activation is likely related to factors presented in pericardial fluid likely involving IL-6 (Zhang et al., 2013; Seo et al., 2018; Steyn et al., 2019), as previously reported (Tang et al., 2018).

### Long-term engraftment after cardiac injection

Cardiomyocytes are considered to be in a terminally differentiated state and lack regenerative capacity. The strategy of cellular transplantation into the infarcted myocardium as a promising means of remuscularizing the dead tissue has been assessed for decades (De Luca et al., 2019). However, there is a growing consensus that the transplanted cells quickly disappear shortly after injection and the beneficial effect of cell transplantation, at best modest and transient, is likely due to paracrine effects (Eschenhagen et al., 2017). In contrast, long-term survival and extensive rebuilding of the damaged myocardium have been achieved by implanting CMs derived from reprogrammed iPS cells or human ESCs in non-human primate hearts (Chong et al., 2014; Shiba et al., 2016). Recently, human iPS-derived Isl-1^+^ ventricular progenitors have shown the ability to stably engraft in the host myocardium and remuscularize chronic scars in a porcine model of MI (Poch et al., 2022). Therefore, it is plausible that the developmental fate of the injected cells largely determines their ability to survive in the host. Our previous studies demonstrated that multipotent, pre-committed pSCs disappeared shortly within the first few days after transplantation and failed to persist within the heart (Tang et al., 2018; Zhu et al., 2022). The cardiac-committed pSCs used in the present experiments, however, showed long-term cardiac retention and stable engraftment in the recipient heart (Figure 5A), suggesting the intrinsic surviving mechanisms are possibly harnessed during the process of cardiac lineage commitment.

Several lines of evidence, albeit highly hypothetical, may help to explain why the committed cells are retained in the myocardium more stably after injection. Physically, the enlarged diameter of the committed cells helps to prevent them from being drained off through the gaps in the damaged blood vessels, as the beating of surrounding cardiomyocytes squeezes the narrow space in the myocardium and adds extra pressure to the transplanted cells. Biologically, the differentiated cells likely undergo a series of metabolic switches that confer unique abilities on the cells of being more resistant to ischemic challenges and forming gap junctions with the host myocardium. The present result serves as a “proof-of-concept” experiment to demonstrate the pericardial source of cardiac-committed cells for cardiac cell-based therapy. Several important issues must be carefully addressed in future studies, including a strategy to fine-tune the molecular process of restricting lineage specification and, importantly, to optimize the protocol for purifying and expanding *in vitro* myogenic progenitors to a clinically relevant scale. As to a time point longer than 4 weeks, it is most likely that the implanted cells are capable of sufficiently persisting in the host myocardium, as after the initial period of survival, the cells have adapted to the cardiac niche, which is relatively mild in the chronic phase. The major barrier that potentially limits cardiac persistency may be the maturation process of the injected cells and the slow development of electrical integration and contractile synchronization to the host tissue. To this point, the electrical property should be carefully investigated in the future. Most important to the highly desired therapeutic goal, the cardiac benefits should be therapeutically explored after the cardiac-committed cells are implanted into an infarcted heart.

In summary, we report here the existence of a quiescent stem cell pool in the pericardial tissue that, in response to inflammatory signaling, gives rise to myogenic progenitors driven by *MyoD* expression. Cardiac transplantation of the cardiac-committed cells yields long-term survival and stable engraftment in the recipient myocardium. Therefore, this finding points to the myogenic progenitors from a pericardial source as an attractive candidate for cardiac cell-based therapy to remuscularize the damaged myocardium. From a clinical perspective, pSC-derived myogenic cells may help settle two major concerns regarding ESCs or iPS-derived CMs: 1) because cardiac-committed pSCs occur naturally, they entail no potential risk of tumorigenesis, and 2) isogenic production of pSCs poses less challenge of immune rejection that requires lifetime immunosuppressive therapy.

## Data Availability

The raw data supporting the conclusion of this article are available in the article and in its online supplementary materials, without undue reservation.
